# The Character Strengths Response: An Urgent Call to Action

**DOI:** 10.3389/fpsyg.2020.02106

**Published:** 2020-08-21

**Authors:** Neal H. Mayerson

**Affiliations:** VIA Institute on Character, Cincinnati, OH, United States

**Keywords:** character strengths, personality, positive psychology, character, strengths, humanity

## Abstract

A model on the role of character strengths in individual, collective, and species success is proffered. It is derived from viewing character strengths from a species perspective as opposed to one of individual differences/personality psychology. The history of the VIA initiative on character science is overviewed, and results to date are summarized in terms of promoting well-being, helping to accomplish aspirational intentions, and allowing the greater good of the collective to grow. “The character strengths response” is described as the response capacities that character strengths may enable for helping us fulfill the human promise of surviving, thriving, and successfully creating a next-generation so that individuals and the collective flourish while also living in harmonious balance with other species. An argument is presented that there is an urgent need for advancing population-wide psychological maturity to be better prepared to navigate the difficult decisions that accompany growing technological powers, and that the character strengths response warrants special attention of research funding to accomplish this imperative.

## Pathways to Human Flourishing

Let’s assume that humans, like other life forms, are endowed with and develop capacities to perpetuate the species. This requires that individuals survive, grow, and produce a successful next generation *without* substantially diminishing or debilitating the greater collective from doing the same. Otherwise, to the degree individuals would use their capacities for self-interest only, without consideration of the impact on others, a species would dwindle, much like the attrition that is experienced in the well-known game of “musical chairs.” Each round of the game reduces the group until there is only one left sitting at the end. *Capacities that promote the individual without diminishing others’ success thereby take on a special importance.* The VIA character strengths (Peterson and Seligman, 2004) are such capacities.

Character strengths enable individual flourishing while at the same time allowing for others to do the same, and sometimes may even enable flourishing in others via inspiration and improved cooperation. They also contribute to resilience in the face of challenges and difficulties, and, as will be described herein, may serve to temper the aggressive and avoidant behaviors that are aroused in the face of perceived threat, commonly known as the fight-or-flight reflex. In this latter role, they can help prevent naturally adaptive defense strategies from deforming into exaggerated misappropriations of violence, and can prevent maladaptive escapism from real problems that are in need of attention. Finally, character strengths can contribute to successfully establishing a next generation. Looking at character strengths from the long-range evolutionary perspective of species success illuminates ways in which character strengths can be deployed robustly to advance the current lives of individuals and their communities.

Thanks to the authors represented herein, and many other pioneering researchers and practitioners, we are expanding our knowledge of these positive psychological characteristics that Drs. Peterson and Seligman (2004) illuminated 20 years ago in their groundbreaking book *Character Strengths and Virtues* and that was presciently recognized by Dr. Howard Gardner as “…one of the most important initiatives in psychology of the past half century” (comment noted with the publication of *Character Strengths and Virtues, 2004*).

## A Young and Urgent Science

The 20 years since the inception of the *VIA Institute on Character’s* initiative on character strength science may seem like a long time, but it’s really not when framed in terms of how long humans have existed and may exist into the future. Modern human beings are estimated to be about 200,000 years old, and it is arguable that we did not begin to apply scientific methods to understand ourselves psychologically until Wilhelm Wundt established the first psychological laboratory 141 years ago in 1879. So, relatively speaking, scientific understanding of our psychological dimension is a very new epistemological pursuit. And, it has only been the past 20 years that a deliberate effort has been going on to create a cohesive scientific knowledge of character strengths. In the big picture of how long our species has existed and how much longer we hope to exist, character strength science is in its infancy. Gott (1993) statistically calculates that, at a 95% confidence interval, we could survive as long as 8 million years and points out that our direct ancestor, Homo Erectus, survived 1.4 million years while Neanderthals lasted only about 300,000 years. While no one knows how long the human species will last, it seems reasonable that we may survive for many more generations to come and that our scientific attention to our psychological nature is young. So, it is only in the last seven one-hundredths of one percent of our life to date that we have been scientifically delving into our psychological nature, and only the past 20 years of those 141 years have we specifically been targeting our strengths of character! Given that we may possibly have *millions* of years yet in front of us, we have many, many years of discovery to which we can look forward.

As you review this volume, you will see we’ve learned a lot in this relatively brief period of time. We are at the very beginning of a long journey ahead which undoubtedly will uncover so much more about the positive personality characteristics with which we are endowed and how they can be utilized to achieve the promise of our human species to actualize our own success without diminishing the same for other people or other living species. It excites the imagination to wonder what the future of this initiative holds in store for us!

Another perspective worth considering as we take this moment in time to reflect on the past 20 years of the VIA character strength science initiative, is one that highlights *the urgency* of this work. People born in the late 19th century came into a world without commercially available motorized vehicles, airplanes or televisions, without home computers, cell phones, or the Internet, without nuclear weapons or remote controlled drone bombers, without genetic engineering capabilities to design life and clone mammals, and with only rudimentary scientific understanding of the psychological dimension of being human. In a mere two or three generations, of the thousands of generations human beings have been around, scientific discoveries have been profoundly rapid and related innovations remarkable. We now live in a world in which:

1. 4.4 billion passengers book airline flights globally, physically connecting everyone on the planet in unprecedented ways.
2. The Internet and cell phones instantly offer connections between 4 billion people globally.
3. Eight mammalian species have been successfully cloned, including sheep, horses, dogs, wolves, and cats.
4. CRISPR technology makes genetic engineering cheaper and faster such that it can be more readily performed, leading to a report in 2018 of the first gene-edited human babies (Ledford, 2019).
5. Artificial intelligence is leading not only to robots that do household chores such as vacuuming, but also that can become “emotionless”, unbiased decision makers when it comes to killing in war, life and death decisions in hospitals, and criminal sentencing.
6. Medical advances now enable the artificial extension of individuals’ lives longer than ever before with life-sustaining medical devices.
7. Military weaponry now includes nuclear weapons which are proliferating and remote-controlled drones capable of bombing and surveillance.
8. Increasing numbers of people have voice-activated assistance devices listening in on them continuously and personal information stored in the public sphere of the Internet.

With these advances in scientific knowledge come ethical decisions requiring wisdom and psychological maturity (Grinbaum and Groves, 2013; New Scientist, 2017). The ethical issues inherent with the above technological advancements may be obvious. With the advent of contagious disease, how do we control transmission given our physical interconnectedness related to global transportation? How do we manage the Internet so as to connect people positively and purvey accurate news and information while controlling it from being a forum for leveraging hate, misinformation, and criminal activity? How will we decide what smart machines to develop and how to deploy them? How will we decide what genetic engineering of plants, animals, and humans will be done despite lack of knowledge of off-target effects? Whose vision of the way things “should be” will guide those decisions? Will we design population control programs and, if so, who will select populations and characteristics to eradicate? How will we resolve our geopolitical conflicts without unleashing untoward damage from weapons of mass destruction and cyberwarfare?

In this paper I suggest that science-based answers to the above questions, and the many like-kind questions that will continue to accrue as we expand our physical science knowledge, reside in the science of human psychology, especially the science of character strengths and virtues. It is asserted herein that the rate of growth in this psychological knowledge is lagging far behind the rate characterizing our physical science knowledge, and that this ever-growing differential defines an emerging “danger gap” worthy of our immediate attention.

**FIGURE 1:**
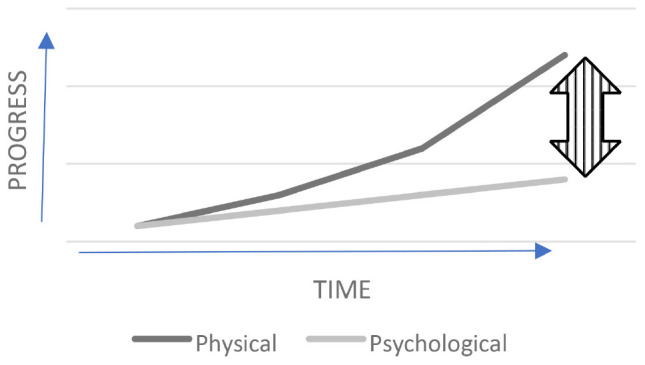


At a conceptual level a graph plotting progress over time in growing our knowledge and capabilities regarding our physical and psychological domains reveals the following. Figure 1 below shows the top line which indicates our growth in knowledge from our physical sciences and the technological innovations resulting from this knowledge. It shows a steep positive slope. This is not a curve plotted with actual data points, but instead only meant to indicate a strong rate of growth. These advancements have been nothing short of amazing. Then, the bottom line shows growth related to understanding ourselves psychologically, and it is relatively flatter, despite advancements in treating psychological suffering and the evidence indicating that a person born today is less likely to die directly at another person’s hands than in the past (Pinker, 2011). Again, the depicted curve is not based on actual data points but only meant to convey a slower rate of growth compared to the top line. How much can we claim to be more mature than our predecessors when it comes to resolving conflict better, living with each other and our environment more harmoniously, and avoiding misappropriations of our aggression, fear, greed, envy, lust, jealousy, and power? To what degree can we claim advancements in our levels of wisdom, transcendence, temperance, humanity, justice, and moral courage? It is the author’s opinion that while incremental improvements might be argued to have occurred over time, they pale in comparison to the growth in physical science-based technologies. This graph reveals that, over time, the gap between our technological advancements and our psychological maturity is ever widening. As this gap grows, the risks inherent in missteps of judgment as regards the application of these technological innovations grow as well, thereby warranting the label of “danger gap” for this growing divergence. In this context it should be noted that, though Gott (1993) estimated an outside range for human survival at 8 million years, his calculation indicates that on the short end our species might only survive another 5,000 years! Without a deliberate effort to advance our psychological capacities much more rapidly, this gap will continue to widen and the danger to us all will grow more ominous. This article aims to make the case that character strengths science is currently the most promising psychological framework for becoming the focal point of intense exploration to narrow the danger gap.

## The VIA Initiative: A New Science of Character Strengths

This journey to create a dedicated scientific effort to understand what’s best in human beings and how we can use those characteristics to build good lives for ourselves and others began in 1999 when Dr. Neal H. Mayerson contacted Dr. Martin E.P. Seligman. The latter was President of the American Psychological Association and was conceiving of a new “positive psychology” to complement the profession’s emphasis on remediating human suffering from psychological disorders. As a practicing clinical psychologist, business entrepreneur, and philanthropist, Dr. Mayerson found Dr. Seligman’s vision compelling. Dr. Mayerson determined to provide the philanthropic support needed to build out what Dr. Seligman conceived to be the “backbone” of this new positive psychology effort, namely the illumination of personal characteristics that propel positive emotions and behaviors and which can be nurtured by social institutions. Good fortune touched this initiative at its beginning when Dr. Christopher Peterson became enamored of this vision enough to sign on to dedicate 3 years of his professional life full-time to co-leading this initiative. Over the next 3 years Drs. Peterson and Seligman spearheaded an unprecedented effort to take a snapshot of what, to that point, was the best thinking on the key psychological characteristics people possess that help us build fulfilling lives and good societies. Parenthetically, though positive psychology quickly became focused on happiness, it can be noted that the originating vision was to build a much broader science, one that looks at the full breadth of what constitutes “a good life” throughout all of the up and down phases we all experience in our lives.

Recognizing the long-term nature of understanding the psychology of character strengths scientifically, Dr. Mayerson established the non-profit organization *VIA Institute on Character* to support this work and pledged to Drs. Seligman and Peterson to have this organization steward the initiative into the future and disseminate its work broadly. The *VIA Institute’s* 3-year project collaborated with 55 psychology scholars and leading figures in the field of positive youth development. The main purposes of the initiative were to lay the intellectual foundation for this new science and to offer the two basic tools any science needs to make progress – namely a *nomenclature* with operational definitions of the main topics of interest, and tools for *measuring* these key constructs in adults and youth. A comprehensive overview of thinking was performed covering the major religions and philosophies from Eastern and Western traditions as well as notions from major works in the humanities and contemporary schema of organizations such as the Boy Scouts and Girl Scouts of America. An effort to capture most of this knowledge into broad categories resulted in the 6 categories now known as the VIA Virtues – wisdom, courage, humanity, justice, temperance, and transcendence. The task then became to detail out these broad constructs with their component elements or dimensions. For example, the construct of Temperance was assigned the component parts of forgiveness, humility, prudence, and self-control. A rigorous process was established using a set of 10 selection criteria for reviewing the multitude of specific characteristics considered as candidates. Among the most important considerations were that the characteristic be elemental in terms of not readily being understood as a combination of other elemental characteristics, that it be universally considered as positively valued across cultures, including some of the most remote indigenous areas on the planet (Biswas-Diener, 2006), and that it be malleable. The resulting 24-character strengths were then conceptually assigned to one of the Virtue categories, with the understanding that as empirical knowledge accumulated and warranted changes in classification or removal altogether, that the scientific evidence would lead the way forward. After articulating the VIA Classification of Character Strengths and Virtues, psychometrically sound measures were developed for use with both adults and youth, known as the VIA Inventory of Strengths (VIA-IS, or VIA Survey) and VIA Inventory for Youth. The totality of this initiative was summarized and published as *Character Strengths and Virtues: A Handbook and Classification* and authored by Drs. Peterson and Seligman (2004) with substantial contributions from the 55 scholars.

After about a year of offering the VIA measurement tools and a free results report online, the response worldwide indicated that the work on character strengths resonated broadly. Along with markers indicating that positive psychology was taking hold, such as national and international conferences, professional journals, book publications, and media coverage, the VIA Institute staffed up to be able to steward this work into the future. Despite the tragically premature death in 2012 of the genius behind this work, Dr. Chris Peterson, the testament to his genius and Dr. Seligman’s has been the continuous growth of this work. At the time of this writing, on average, a person takes the VIA Survey every 10 seconds of *every minute* of every hour of the year, and that rate has been accelerating every year! It has been translated into over 41 languages, and over 700 research articles have been published on the VIA character strengths, their classification, and measurement (Via Institute on Character, 2020a, b). Thought leading books have translated this emerging science into practical guides for coaches, mental health professionals, managers and educators as to how they can apply character strengths in their work (Niemiec, 2018) and how laypeople can develop any of their 24-character strengths to improve their lives (Niemiec and McGrath, 2019).

## What We’Ve Learned About Character Strengths

In terms of the basic aims of establishing a nomenclature for building a new scientific effort along with tools of measurement, we have learned the following. First, as Peterson and Seligman initially presented, the VIA Classification was intended to be an intellectual framework to begin generating meaningful scientific activity despite it admittedly being an imperfect beginning point. Given the above mentioned volume of research articles that have been published, the VIA Classification has been doing its job to initiate what promises to be a long road of scientific inquiry. And, despite some debate on its merits (Banicki, 2014; McGrath, 2018; Snow, 2018), the Classification has been largely supported by empirical research (McGrath et al., 2018; Ruch et al., 2019). There has not yet been an accumulation of compelling evidence to suggest the need for modifying the Classification [but see initial efforts by Ruch and Proyer (2015)].

Regarding measurement, efforts at continuous improvement have been ongoing, with the most recent suite of measurement tools being released in 2018 by the VIA Institute. Results to date indicate that we are able to measure the 24-character strengths and the six virtues in accordance with conventional psychometric standards (see technical report for the suite of VIA assessments, McGrath, 2019).

**FIGURE 2:**
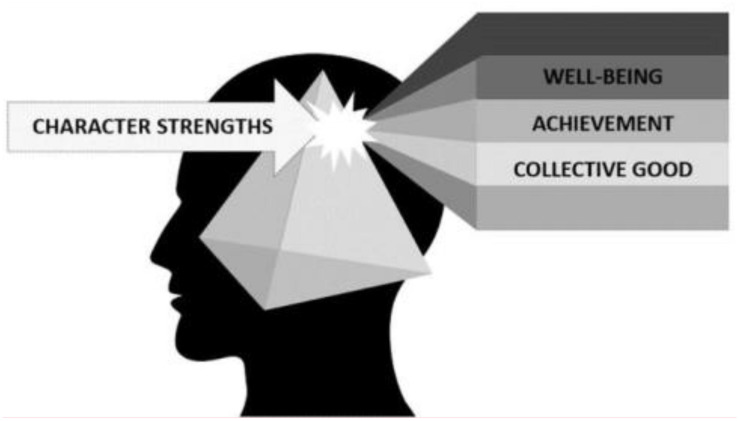
Utility of character strengths.

As the beam of 24-character strengths has been passed through the prism of scientific inquiry, three notable refractions have become evident, as described below and depicted in Figure 2.

The first refraction has to do with well-being. Character strengths define essential aspects of our personal identity, and well-being is related to congruence between who we are and what we do. Robust associations between character strengths expressions and a variety of broad constructs indicative of well-being have been found (Wagner et al., 2019), and positive emotions that are markers of well-being have been associated with character strengths (Güsewell and Ruch, 2012). Since the character strengths have been selected based on being universally valued, which has been empirically supported (McGrath, 2015), conceptually they are expected to be reinforced generally by others when we display them. This leads to feelings of acceptance and appreciation that also are important contributors to well-being. In addition to being externally reinforced, character strengths are intrinsically fulfilling – we feel good when we recognize them in ourselves and when we express them. Thus, even in environments that are not supportive of a signature strength, expressing the strength can nonetheless be fulfilling. For example, a person might have creativity as an important element in their identity, and, despite living in an environment that does not encourage nor reward creativity, the person might nonetheless find meaningful fulfillment from privately creative behavior. Peterson and Seligman (2004) described this as being akin to the Aristotelian notion of eudaemonia in which actions are intrinsically fulfilling in and of themselves, despite whether or not they produce valued outcomes.

A second refraction revealed by character strengths science has to do with their instrumentality. Character strengths direct us into meaningful and engaging activities to which we aspire, both as individuals and in relationships with others. They help us succeed in what we aim to accomplish. They fuel productivity. First, the fact that the character strengths were selected based on being positively valued throughout the ages and across cultures as pathways to “a good life” inherently aligns them well as catalysts of valued outcomes. Seligman has described in his PERMA model of human flourishing (2012) how character strengths are important pathways to each of the elements of flourishing: positive emotions, intrinsic engagement, positive relationships, sense of meaning and purpose, and accomplishments. And, character strengths have been associated with productivity at work, in the classroom, and in personal goal achievement (Linley et al., 2010; Lavy and Littman-Ovadia, 2016; Weber et al., 2016). They help us achieve what we want to *do* in life, and as such they promote “well-doing” (Lottman et al., 2017).

These two refractions suggest that character strengths function as psychological connective tissues, engaging who-we-are with what-we-do so as to produce fulfilments. Findings showing associations between character strengths and engagement in jobs (Lavy and Littman-Ovadia, 2016; Bakker et al., 2019), classrooms (Park and Peterson, 2008, 2009; Wagner and Ruch, 2015), and relationships (Veldorale-Brogan et al., 2010; Guo et al., 2015; Kashdan et al., 2017) support this insight.

The third refraction, and one that differentiates character strengths from many other personality characteristics such as neuroticism or aggressiveness, is that while they are advancing the interests of the individual, they are not diminishing others’ opportunities to do the same. They drive the non-zero behaviors that play such an important role in human progress (Wright, 2000), and may even promote expression of character strengths in others. Early evidence is coming together to support this latter assertion. Haidt (2003) described the phenomenon of “elevation” as a positive emotion experienced upon witnessing virtuous acts, and that it motivates individuals to act more virtuously themselves. Numerous studies now show this emotion of elevation, which is a dimension of the character strength known as appreciation of beauty, leads people to not only *be motivated* toward goodness but to *actually behave* prosocially (e.g., Schnall and Roper, 2011). And, while emotional contagion, both negative and positive, has been reported for quite some time (Hatfield et al., 1994), more recently *positive* emotional contagion has been noted to spread in surprising ways through real life social networks (Fowler and Christakis, 2008) and even to transfer through virtual social networks such as Facebook (Kramer et al., 2014). In sum, this third refraction emphasizes how character strengths hold the substantial potential for contributing to the collective good.

## Toward a Theory: The Character Strengths Response

At the outset of the VIA initiative on character strengths and virtues there was a retreat held in Glasbern, Pennsylvania with a diverse group including leading practitioners in the field of positive youth development, professionals who played a significant role in developing the DSM diagnostic manual of mental illnesses, psychologists, philosophers, educators, and a representative from the field of botany familiar with taxonomic development. At that meeting it was underscored that a true taxonomy requires an underlying theory regarding its components. Recognizing that no consensually agreed upon theory of character strengths and virtues existed at the time, it was decided that the appropriate aspiration was a *classification* as opposed to a true taxonomy. Hence, the VIA *Classification* of Character Strengths and Virtues. At this point, 20 years later, there still is no consensually agreed upon theory, but there is thinking that is leading toward such a theory. While character strengths science emerged mainly from the perspective of individual differences/personality psychology, further insights about them can be gleaned from an evolutionary perspective.

As described at the outset of this article, species succeed by having their individuals *survive*, *grow*, and *successfully establish a next generation*. A primary focus of positive psychology has been thriving, which can be understood as the growing toward one’s positive potentials. While a secondary focus has looked at resilience in the face of difficulties, exploration of the role of character strengths in producing resilient and aspiring offspring is in its early stages, and exploration of their role in our repertoire of survival instincts and strategies has not yet begun.

**FIGURE 3:**
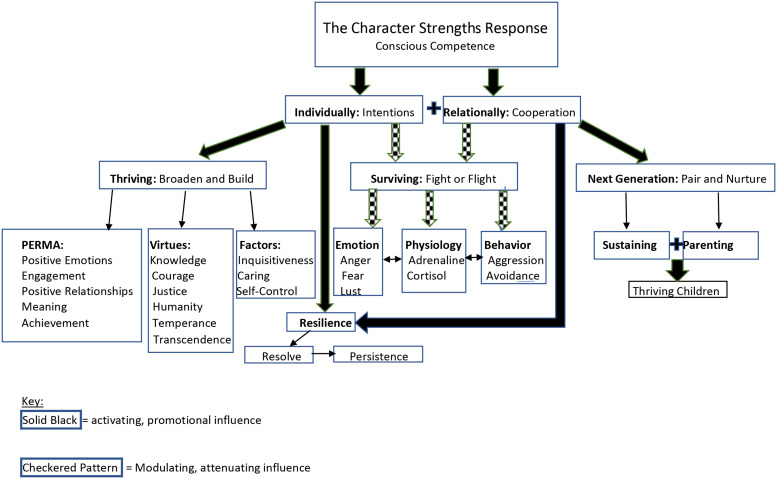
A model of character strengths purposes.

Figure 3 below summarizes a model of the role of character strengths that emerges from the perspective of species success, and that will be fleshed out in the sections to follow. It highlights “the character strengths response” which can be understood as our capacity to respond to life circumstances with our character strengths so as to optimize individual and collective success, and a response that can be developed from unconscious competence, or incompetence, into conscious competence. This model shows a facilitating role of character strengths toward thriving, resilience, and successful creation of a next generation through their influences on individuals’ intentions and cooperative relationships. And it shows an attenuating influence on our aggression and avoidance impulses that are fundamental to our innate survival response. This attenuating effect over the primitive fight-or-flight *survival* reflex is proposed to keep those instincts from running amok into maladaptively excessive aggression and avoidance, thereby giving promise to promoting more peaceful cohabitating.

### Thriving

Early in the advent of positive psychology Frederickson (2001) described the “broaden and build” theory. It describes how during times of threat our attention narrows to focus on the danger at hand, “negative” emotions such as fear and anger are aroused, and then behavioral responses to either fight or flee get set in motion reflexively, but, when threat is less present, and positive emotions have more breathing room to emerge, they produce a broadening of attention allowing new learning and building of capacities for future surviving and thriving. This theory has garnered popularity as it is a useful way of thinking not only about the roles of positive emotions, but more generally the processes underlying positive psychological growth. Growth is promoted by a broadening of our attentional bandwidth that results in expanding opportunities for building new skills. Might it be that when our sense of threat is lower, and our sense of safety is higher, that our character strengths also move to the forefront to help us grow toward our highest potential, individually *and* collectively? It could be especially during these windows of perceived safety that we broaden our capacities to survive and thrive by “building-up” lesser strengths and “building-upon” already prominent strengths. Advancement in building-upon strengths can be accomplished by improving our expertise in minimizing overuse and underuse and in finding the golden mean of expression – the right strength in the right proportion in the right circumstance. This hypothesis has yet to be tested.

Soon after Frederickson introduced her theory of positive emotions, Seligman (2002a) introduced his model of “authentic happiness” which later was modified into his PERMA model of human flourishing Seligman (2012). The latter model describes key elements contributing to flourishing, namely: positive emotions and relationships, engaging and meaningful activities, and achievement. This describes *what* we can direct ourselves to *broaden* and *build* in order to create good lives for ourselves and others. Character strengths are posited as important pathways to each of these elements and we can deploy them to build positive relationships, positive emotions, and the other elements in this model. For example, curiosity can lead toward engagement, positive relationships, and positive emotions. Developing *conscious competence* in deploying our character strengths to build out the key elements of flourishing is a way for humanity to advance toward its positive potential. Conscious competence results when automatic responses that occur subconsciously are made conscious and their activation becomes deliberate and practiced. For example, my automatic inclination toward critical thinking can be made conscious and thereby managed better so that it is not my first response in situations where kindness or love, for example, might be more appropriate.

More recently McGrath (2018) published compelling findings from extensive factor analytic studies indicating three primary factors appearing in VIA’s world database of character strengths. He named these factors “inquisitiveness”, “caring”, and “self-control” and described the confluence of this structure with prior conceptual models of virtue. This empirical approach suggests an underlying structure of the 24-character strengths and provides another perspective of *what* specific capacities we can direct our use of character strengths toward in order to broaden and build. Namely, by broadening our capacities for *acquiring knowledge*, establishing *positive relationships* of caring, and *managing impulses* in service of successful performance and goal attainment, we build our pathways to thriving. As individuals, and as a collective, our lives improve as we broaden and build ourselves in each of these three domains.

Figure 3 depicts the above models, along with the original six virtues model of the VIA Classification, as examples of targets toward which we can direct our character strengths in order to help us broaden and build toward thriving. These examples are not definitive nor exhaustive, and the model described in Figure 3 is agnostic to theories of thriving. Instead, this model simply asserts that, as key elements of thriving are illuminated, character strengths can likely help us pursue these elements successfully.

### Surviving/Resilience

While clarity is growing that character strengths are psychological capacities that help us grow and thrive, their utility extends further. As Seligman initially postulated, a focus on positive characteristics not only promises to lead us to greater flourishing, but, at the same time, might illuminate ways to prevent problems and become more resilient in facing them (Seligman and Csikszentmihalyi, 2000; Seligman, 2002b). Common experience teaches us that character strengths can be forged in the crucible of the stresses and strains we encounter in life, and that they can be instrumental in getting us through those challenges. Niemiec (2019a, b) elaborated on the roles character strengths can play in resilient coping with stress, while Harzer and Ruch (2015) found that character strengths were connected with improved coping with work stress and that they decreased the negative effects of experienced stress. Shoshani and Slone (2016) found that character strengths were associated with resilience among adolescents exposed to lengthy periods of war, terrorism, and political conflict. Peterson et al. (2008) even reported the provocative finding that the *more* traumatic events an individual reported the *higher* their character strength scores were. And, Chopik et al. (2020) found stability of character strengths pre and post military deployment.

And we can look at virtually any crisis that communities have experienced and recount how character strengths came forth to not only help ourselves weather the storm, but also to reach out and help others. Peterson and Seligman (2003) compared scores for people who completed the VIA Survey online 2 months before the September 11, 2001 terrorist attack with those individuals who completed the VIA Survey in the 2 months after the attack, and found seven character strengths showed increases that endured 10 months after the attack. Of course, limitations of their methodology render the results suggestive of actual changes in character strengths (Lamade et al., 2020) and not indicative. Or, consider stories of *righteous gentiles* and *upstanders* during the Holocaust, when people exercised bravery, kindness, creativity, fairness and most of the other character strengths, despite great peril to themselves (Paldiel, 2007). In the ordinary course of life, many of us discover how strengths such as hope, perspective, and compassion help us through tough losses, such as the loss of a loved one, as well as helping us cope with disappointments of various intensities. As stated by Niemiec (2019), “character strengths offer an important role in buffering, reinterpreting, managing, and transforming the adversities and problems of life”, and he notes that all 24 strengths have been linked scientifically at some point with resilience. This enables us to emerge from challenges with strengths intact for moving forward positively.

Examining character strengths from the perspective of species success points to how, in addition to helping us be resilient, character strengths may play a role in helping us modulate over-reaction of our fight-or-flight response, exemplified by levels of aggression or avoidance out of proportion to the actual threat at hand or misapplied to innocent targets. This fight-or-flight reflex has physiological, behavioral, and emotional components, which activate our motivation and capacity to effectively fight or flee (Russel and Lightman, 2019). Fear, anger, and lust, as examples, fuel aggressive behavior, as does activation of physiological bursts of cortisol and adrenaline. Emotions, cognitions, and physiological responses supporting fighting or fleeing surge with mutually amplifying effects on each other. We get amped up to deal with danger. Unchecked, these physiological, cognitive, and emotional fuels can explode into grotesque aggression that is misdirected and produces unnecessary collateral damage that is not only maladaptive but also morally offensive. Again, consider the Holocaust in which ordinary Germans, feeling economically and culturally vulnerable, came to *inaccurately* perceive a highly *distorted level of threat misattributed* to the Jewish race, unleashing aggressive capacities designed for adaptive protection that deformed into the vulgarities which mark this darkest period in modern human history.

The proposed modulating effects of character strengths may enable us to defend ourselves with wisdom, courage, temperance, humanity, justice and transcendence. For example, it has been found that the strengths of honesty, persistence, and love moderate aggression (Park and Peterson, 2008). Many of us can relate to times when our anger response ignited and we felt adrenaline rushing into our body, only to have both impulses moderated by finding perspective or love, as examples, thereby bringing us to a more measured and appropriate response. Future research and theory development into the role of the character strengths response in modulating our survival instincts is needed to determine the conditions under which, and the degree to which this modulation can occur.

### Creating the Next Generation

At a species level, successfully creating a next generation depends on establishing and sustaining positive relationships that produce offspring and bring necessary resources to rearing them to be successful in life. Setting aside issues about the relevance of whether children are reared by biological parents or others, or single parents, it makes sense that the challenges of child-rearing are substantial and that having more resources of emotional, financial, cognitive, and relational supports furthers successful child-rearing compared with the resources of one person alone (Amato, 2001). As such, positive relationships between healthy people sharing parenting responsibilities can be expected to further the species-level goal of successful reproduction. This points us to look at the role of character strengths in sustaining positive relationships with others who have roles in raising children.

Guo et al. (2015) reported that marriages with greater levels of satisfaction were associated with children having greater levels of character strengths. And Waters and colleagues have found that strengths-based parenting has a positive effect on child academic achievement, stress coping, and life satisfaction (Waters, 2015a, b; Waters et al., 2019). Kashdan et al. (2017) studied a community sample of couples living together in a romantic relationship for at least 6 months and found that relationships were stronger along a number of dimensions when partners recognized and appreciated character strengths in one another. And, the role of character strengths in child rearing is further highlighted by findings that parental well-being is associated with improved child outcomes (e.g., Dumas and Wekerle, 1995; Leung and Slep, 2006) and that character strengths are positively associated with well-being.

So, character strengths can contribute to the well-being of parents, to the positive relationship of parents with one another and others involved in child-rearing, and to raising children with improved health and well-being. Much more research on this topic is needed.

### Cooperating

The character strengths response model depicted in Figure 3 indicates that character strengths exert their influence through us as individuals, but also posits that they may help us improve how we work and live *together*. Cooperation not only enables effective group efforts and individual achievements (Grant, 2013), but, based on the social norm of the reciprocity principle (Gouldner, 1960), it also presents individuals with opportunities for personal growth. Hence, the oft asserted pragmatic adage, “Personal success is not so much determined by *what* you know as much as by *who* you know”, and the aphorism “What goes around comes around.” Doors of opportunity get opened, directly and indirectly, by people with whom we have positive relationships.

Economic games are analogs of real-life situations that are used to study the factors determining competition and cooperation. In these games one player can compete for his/her self-interest at the expense of others or by settling for a lesser reward, or they can choose to cooperate with others and get a greater reward but at the risk that others will not also choose to cooperate in good faith (trust), which would thereby undermine the promise of a higher payoff. These well-researched experimental paradigms offer a method for exploring how character strengths may impact cooperation and competition. Pioneering researchers have begun to look at individual differences in character strengths to see how they impact decisions to compete and cooperate with one another in economic games. These early explorations indeed suggest that knowledge of individuals’ character strengths can add power to predicting the degree to which subjects will be cooperative and caring as opposed to selfish and unkind when presented with dilemmas involving economic gain (Ruch et al., 2017; Jordan and Rand, 2018). These early findings are encouraging for further research of this kind.

And, early work has begun looking at the implications of character strengths for understanding how employees cooperate in work teams to influence productivity and quality of experience. After reviewing models of team roles and functions (e.g., Belbin, 2012), the author articulated seven roles that occur as employees work together in teams. These include the tasks (and roles) of: creating ideas (idea creator), gathering information to consider in deciding the value of the idea (information gatherer), considering the evidence in making a decision (decision-maker), implementing the decision (implementer), persuading others of the merits of the new program/product (influencer), managing relationships along the way (relationship manager), and keeping energy going throughout the project (energizer). Willibald Ruch and his colleagues established a reliable measure of these roles as self-reported by employees, established the validity of this seven function classification, developed an algorithm that utilized all 24 VIA character strength scores for each individual, and found that this algorithm predicted which roles individuals reported as enjoying and performing well (Ruch et al., 2016, 2018). They then looked at actual work teams and discovered that balance across the team in these character strength related roles predicted self-reported and supervisor-reported measures that included quality of team experience and aspects of performance (Gander et al., 2018, 2020). For example, self-rated work satisfaction and teamwork quality were predicted by a number of character strengths, most strongly teamwork and love.

Character strengths hold promise for shedding light on how we might work and live together better as a result of understanding our own and each other’s character strengths profiles.

## Future Research

Research, practice, and modeling to date on the *VIA Classification* suggest the following potential lines of research for consideration:

### Thriving

While association between character strengths and thriving has been a robust finding, what is now needed are more intervention studies to establish causal relationships.

1.
  (a) Instrumentality: Does deliberate application of character strengths to aspirational goals improve goal achievement? Which specific strengths and strengths combinations are best for achieving which specific outcomes?
  (b) Well-being: Does well-being improve as one expands the degree to which their life activities resonate with signature strengths (character strengths especially important to personal identity)? How is well-being impacted by overuse of character strengths?
  (c) Collective good: Is there a character strengths contagion phenomenon – i.e., does observation of character strengths expression in others increase the likelihood of character strength expression by observers?

### Surviving

1.
  (a) Resilience: What determines whether character strengths are activated in the midst of challenges and struggles, and, and later as they move past the challenge? Does greater awareness and development of character strengths prior to a crisis result in greater resilience through the crisis?
  (b) Modulating the fight-or-flight response: What determines the degree to which character strengths become coupled with angry and aggressive impulses to appropriately modulate them? What are the limiting factors of how much character strengths can modulate aggression?

### Child-Rearing

1.
  (a) How can parents leverage their character strengths to establish and maintain supportive relationships for child-rearing?
  (b) How can parent knowledge of their own and each other’s character strengths improve parenting?
  (c) Does deliberate effort to nurture children’s character strengths lead to better child outcomes?
  (d) Can character strength related activities buffer against negative childhood experiences and promote positive adult functioning, as has been noted with the impact of positive childhood experiences on adverse childhood experiences (Bethell et al., 2019)?

### System Dynamics

The intercorrelations of the character strengths suggest that they may interact dynamically with one another as opposed to asserting their influences individually (Breen et al., 2010).

1.
  (a) How do combinations of character strengths and profiles of character strengths impact behavioral expressions?
  (b) Is there a “towing principle” in which top strengths can help pull forward lesser strengths?

### Interpersonal Dynamics

1.
  (a) Are successful romantic relationships characterized by some degree of similarity, thereby creating a bond of belonging, *along with* some degree of complementarity which stimulates growth and expands the capacities of the coupled unit?
  (b) Can conflict be resolved by learning to see the other person’s offensive behavior in terms of their character strengths (Mayerson, 2016)?
  (c) Expansion of research using the economic games research paradigms.
  (d) Can training couples to appreciate each other’s character strengths improve distressed relationships?
  (e) Can corporate and governmental decision-making teams improve based on deliberate consideration of character strengths in team composition?

### Contextualizing

Character strengths are expressed in contexts and therefore we need to understand better how context elicits character strength responses.

1.
  (a) How do context characteristics determine which character strengths are likely to be elicited – e.g., public vs. private, strangers vs. close relationships, work vs. social.

### Strengths Spotting

1.
  (a) What cues do we use to identify character strengths in others?
  (b) In perceiving others’ strengths, do we have perceptual or attributional biases, such as the self-confirming tendency to see strengths that are most prominent in ourselves?

### Development Across Lifespan

We need longitudinal studies of the natural development of the character strengths from birth onward to uncover if there are critical periods for the development of certain ones, and what processes seem most influential in setting courses of development.

1.
  (a) Do character strengths that have presumably lower genetic loading and that are highly socialized (socialized self) operate differently in a person’s life than those that may be presumed to have high genetic loading and are highly socialized as well (authentic self)?
  (b) Do some character strengths naturally develop at different points in life (e.g., does spirituality emerge later than curiosity)?

In all of these lines of inquiry, while we tend to look initially for broad linear effects, we also need to progress to studying *specific* effects, and ones that are *non-linear*. Regarding specific effects, we need to learn more about which strengths are best at playing what roles in which contexts. As an example, perseverance has been suggested to play the most important role for work performance (Littman-Ovadia and Lavy, 2015). And, Shoshani and Slone (2012), in studying transition of students from middle school, found that temperance strengths were central in predicting *school performance and well-being*, while interpersonal strengths best predicted *social functioning* at school. And, signature strengths at work influenced *behavioral* outcomes while the “happiness strengths” of zest, gratitude, love, curiosity, and hope had the greatest influence on *psycho-emotional* outcomes such as meaning and satisfaction (Littman-Ovadia et al., 2016). A summary of specific effects that have already been published would be a good first step.

With regard to non-linear effects, Gander et al. (2020) found that certain team roles, that are differentially predicted by character strengths profiles, have a quadratic relationship with team performance, meaning too little of that role in a team hurts performance and too much of that role also hurts performance. In this same study they did not find any quadratic relationships between specific character strengths representations on a team and team performance. That being said, the question about whether there can ever be too much of a character strength remains an open one. Studying strengths “overuse” (Niemiec, 2019b) might require quadratic analysis of strengths-in-specific-situations. For example, too little curiosity or love of learning in a student might harm performance and too much might also be detrimental by sending the student down tangential paths of interest that are off-task from learning the course-specific content. Shin and Grant (2020) found an inverted “U” shaped curve relationship between procrastination and creativity.

Finally, future research might consider the work of Todd Rose (2015) in which he points out the potential advantages of utilizing non-ergodic research methods. He explains that conventional social science statistics and methods are based on ergodic theory that focuses on group averages, and that the underlying assumptions of this approach require that *one can only use group averages to infer predictions about individuals if* a.) every member of the group is identical, and b.) every member of the group will remain the same in the future. These criteria obviously do not apply to human research subjects, yet we do tend to translate findings from group studies to individual applications. Non-ergodic approaches might provide new insights to complement what gets uncovered with the conventional ergodic methods used in social science. A non-ergodic approach might be especially applicable to studying the natural course of development of the character strengths across the lifespan as well as changes in character strengths, especially signature strengths, over time and conditions (Wright and Zimmerman, 2019: Beck and Jackson, 2020).

## Closing the “Danger Gap”: A Call to Action

Character strengths science has revealed character strengths as psychological levers that a.) can influence a broad range of universally valued outcomes, b.) can be studied scientifically, and c.) resonate broadly with the lay public. They are readily understood, measured, and utilized. Research findings to date suggest they hold great promise to be able to be deployed to simultaneously enable good lives for ourselves and others, help prevent excessive violence and escapism, and help us successfully parent next generations. Because we are living in a world in which we have ever-increasing technological powers that require wise decision-making, focusing on character strengths science takes on an immediate urgency. Certain errors in judgment as to the application of our technologies can have devastatingly negative impacts. We stand on a precipice that is only getting more and more unstable as time marches on, and advancing our collective psychological maturity is an imperative.

Character strengths science holds the promise of accomplishing our immediate, mid-term, and long-term goals. Our immediate goal is to make the most of our individual lives while not unnecessarily diminishing others’ capacities for doing the same. Our mid-term goal is to set the stage for our next generation to advance further in constructing good lives for themselves and each other. And, our long-term goal is to set in motion a trajectory to enable successive generations to keep advancing further and further toward fulfilling the ultimate human promise.

The immediate call to action is to increase allocation of financial resources to character strengths science so that we can discover their full potential. As described herein, given the need for broadscale psychological growth and the potential for the character strengths response to have broad ranging impacts to set us on a positive course individually and collectively, character strengths science stands at the forefront in terms of warranting further funding support. Pursuing answers to the research questions above will directly position us better to reduce the danger gap described herein which has an immediacy about it. Fortunately, even modest adjustments in existing financial resources will make a huge difference. The author appeals to funders to prioritize just a fractional amount of their budgets to character strengths science, since such an allocation from various sources can provide an immense boost to this important research area to help discover the degree to which these psychological tools can help as much as the early research returns suggest they might.

Secondly, we now know enough about ourselves as psychosocial beings to warrant immediate widespread application of this knowledge in our social institutions. Schools have a critical role to play (Linkins et al., 2015). It is realistic to imagine an upcoming generation that has been inculcated each and every year with advancing knowledge about social and psychological resilience and wellbeing, and how to deploy our full range of psychological capacities to both flourish and be appropriately protective and successful through difficulties and crises. It is time now for character strengths science to become part of the core sciences and humanities curricula. Beyond schools, one can envision that organizations of all types will come to leverage the strengths of their employees and members as a fundamental aspect of organizational culture (Adler, 2008). Businesses can become places where employee’s strengths are magnified and then refracted into society through their personal lives.

It is now time to be determined about nurturing widespread positive psychological mindedness, in particular our capacities for virtuousness. As it has been noted that our brains are wired to pay greater attention to negative events than positive ones (Ito et al., 1998; Vaish et al., 2008; Soroka et al., 2019), so it may also be that the impulse driving our “*character strengths response”* is considerably weaker than our survival response. This means that we should expect that efforts to strengthen this response will need to be especially substantive and sustained. We need to appropriate much greater efforts than we have to date.

Our *human* promise is rooted in the broad range of positive capacities we possess and are able to grow to sustain our own longevity while living in respectful balance with other living species. Character strengths are important endowments we possess for delivering this promise and it has become urgent that we marshal our resources to advance our understanding of them. They are tangible psychological levers that we can operate to develop the grit and the grace we need currently and into the future.

This generation, and more so the one that follows, and the one that follows that, can develop the “character strength response” to the point of becoming a powerful enough response to position us better to manage wisely the powers we keep amassing. The tools are in our hands, and the time is now, to build the fulcrum around which humanity can begin tipping toward its highest promise.

## Author Contributions

The author confirms being the sole contributor of this work and has approved it for publication.

## Conflict of Interest

The author declares that the research was conducted in the absence of any commercial or financial relationships that could be construed as a potential conflict of interest.

## References

[B1] Adler S. (2008). “Global business as an agent of world benefit: new international business perspectives leading positive change,” in *Handbook of Research on Global Citizenship*, eds Scherer A. Palazzo G. (Northampton, MA: Edward Elgar Publishing).

[B2] Amato P. R. (2001). Children of divorce in the 1990s. An update of the Amato and Keith (1991) meta-analysis. *J. Fam. Psychol.* 15 355–375. 11584788 10.1037//0893-3200.15.3.355

[B3] Bakker A. B. Hetland J. Olsen O. K. Espevik R. (2019). Daily strengths use and employee well-being: The moderating role of personality. *J. Occupat. Organ. Psychol.* 92 144–168. 10.1111/joop.12243

[B4] Banicki K. (2014). Positive psychology on character strengths and virtues. A disquieting suggestion. *New Ideas Psychol.* 33 21–34. 10.1016/j.newideapsych.2013.12.001

[B5] Beck E. Jackson J. (2020). Idiographic traits: a return to Allportian approaches to personality. *Curr. Direct. Psychol. Sci.* 29:096372142091586.

[B6] Belbin R. M. (2012). *Team Roles at Work.* New York, N.Y: Routledge.

[B7] Bethell C. Jones J. Narangerel G. (2019). Positive childhood experiences and adult mental and relational health in a statewide sample. *J. Am. Med. Assoc. Pediatr.* 173:e193007. 10.1001/jamapediatrics.2019.3007 31498386 PMC6735495

[B8] Biswas-Diener R. (2006). From the equator to the North Pole: A study of character strengths. *J. Happ. Stud.* 7 293–310. 10.1007/s10902-005-3646-8

[B9] Breen W. E. Kashdan T. B. Lenser M. L. Fincham F. D. (2010). Gratitude and forgiveness: Convergence and divergence on self-report and informant ratings. *Pers. Individ. Differ.* 49 932–937. 10.1016/j.paid.2010.07.033 20871779 PMC2943233

[B10] Chopik W. Kelley W. Vie L. Bonett D. Lucas R. Seligman M. (2020). Development of character strengths across the deployment cycle among U.S. army soldiers. *J. Pers.* 1–12. 10.1111/jopy.12564 32453864

[B11] Dumas J. Wekerle C. (1995). Maternal reports of child behavior problems and personal distress as predictors of dysfunctional parenting. *Dev. Psychopathol.* 7:465479.

[B12] Fowler J. Christakis N. (2008). Dynamic spread of happiness in a large social network: longitudinal analysis over 20 years in the Framingham Heart Study. *BMJ* 337:a2338. 10.1136/bmj.a2338 19056788 PMC2600606

[B13] Frederickson B. (2001). The role of positive emotions in positive psychology: the broaden-andbuild theory of positive emotions. *Am. Psychol.* 56 218–226. 10.1037/0003-066x.56.3.218 11315248 PMC3122271

[B14] Gander F. Gaitzsch I. Ruch W. (2020). The relationships of team role- and character strengths-balance with individual and team-level satisfaction and performance. *Person. Commun.* 10.3389/fpsyg.2020.566222 PMC7734085 33329199

[B15] Gander F. Ruch W. Platt T. Hofmann J. Elmer T. (2018). Current and ideal team roles: Relationships to job satisfaction and calling. *Transl. Issues Psychol. Sci.* 4 277–289. 10.1037/tps0000165

[B16] Gott J. (1993). Implications of the Copernican principle for our future prospects. *Nature* 363 315–319. 10.1038/363315a0

[B17] Gouldner A. W. (1960). The norm of reciprocity: a preliminary statement. *Am. Sociol. Rev.* 25 161–178.

[B18] Grant A. (2013). *Give and Take: Why Helping Others Drives Our Success.* New York, NY: Penguin Books.

[B19] Grinbaum A. Groves C. (2013). “What is “Responsible” about responsible innovation? understanding the ethical issues,” in *Responsible Innovation (2013)*, eds Owen R. Bessant J. (New York, NY: John Wiley & Sons, Ltd), 119–142. 10.1002/9781118551424.ch7

[B20] Guo J. Wang Y. Liu X. Y. (2015). Relation between marital satisfaction and character strengths in young people. *Chin. Ment. Health J.* 29 383–388.

[B21] Güsewell A. Ruch W. (2012). Are only emotional strengths emotional? character strengths and disposition to positive emotions. *Appl. Psychol.* 4 218–239. 10.1111/j.1758-0854.2012.01070.x 26286979

[B22] Haidt J. (2003). “Elevation and the positive psychology of morality,” in *Flourishing: Positive Psychology and the Life Well-Lived*, eds Keyes C. L. M. Haidt J. (Washington DC: American Psychological Association), 275–289. 10.1037/10594-012

[B23] Harzer C. Ruch W. (2015). The relationships of character strengths with coping, work-related stress, and job satisfaction. *Front. Psychol.* 6:165. 10.3389/fpsyg.2015.00165 25767452 PMC4341515

[B24] Hatfield E. Hatfield C. Cacioppo J. Rapson R. (1994). *Emotional Contagion.* New York, NY: Cambridge University Press.

[B25] Ito T. A. Larsen J. T. Smith N. K. Cacioppo J. T. (1998). Negative information weighs more heavily on the brain: The negativity bias in evaluative categorizations. *J. Pers. Soc. Psychol.* 75:887. 10.1037/0022-3514.75.4.887 9825526

[B26] Jordan M. Rand D. (2018). The Role of Character Strengths in Economic Decision-Making. *Judgment Decis. Mak.* 13 382–392.

[B27] Kashdan T. Blalock D. Young K. Machell K. Monfort S. Mcknight P. (2017). Personality strengths in romantic relationships: measuring perceptions of benefits and costs and their impact on personal and relational well-being. *Psychol. Assess.* 30 241–258. 10.1037/pas0000464 28383929

[B28] Kramer A. Guillory J. Hancock J. (2014). Experimental evidence of massive-scale emotional contagion through social networks. *Proc. Natl. Acad. Sci.* 111 877–890. 10.1073/pnas.1320040111 PMC4066473 24889601

[B29] Lamade R. Jayawickreme E. Blackie L. McGrath R. (2020). Are sequential sample designs useful for examining post-traumatic changes in character strengths? *J. Positive Psychol.* 15 292–299. 10.1080/17439760.2019.1610481

[B30] Lavy S. Littman-Ovadia H. (2016). My better self: Using strengths at work and work productivity, organizational citizenship behavior and satisfaction. *J. Career Dev.* 44 1–15. 10.1177/0894845316634056

[B31] Ledford H. (2019). CRISPR babies: when will the world be ready?. *Nature* 570 293–296. 10.1038/d41586-019-01906-z 31217610

[B32] Leung D. Slep A. (2006). Predicting inept discipline: The role of parental depressive symptoms, anger, and attributions. *J. Consult. Clin. Psychol.* 74 524–534. 10.1037/0022-006x.74.3.524 16822109

[B33] Linkins M. Niemiec R. Gillham J. Mayerson D. (2015). Through the lens of strength: a framework for educating the heart. *J. Posit. Psychol.* 10 64–68. 10.1080/17439760.2014.888581

[B34] Linley P. A. Nielsen K. M. Gillett R. Biswas-Diener R. (2010). Using signature strengths in pursuit of goals: Effects on goal progress, need satisfaction, and well-being, and implications for coaching psychologists. *Int. Coach. Psychol. Rev.* 5 6–15.

[B35] Littman-Ovadia H. Lavy S. (2015). Going the extra mile: Perseverance as a key cgaracter strength at work. *J. Career Assess.* 24 240–252. 10.1177/1069072715580322

[B36] Littman-Ovadia H. Lavy S. Boiman-Meshita M. (2016). When theory and research collide: examining correlates of signature-strengths use at work. *J. Happiness Stud.* 18 527–548. 10.1007/s10902-016-9739-8

[B37] Lottman T. Zawaly S. Niemiec R. M. (2017). “Well-being and well-doing: Bringing mindfulness and character strengths to the early childhood classroom and home,” in *Positive Psychology Interventions in Practice*, ed. Proctor C. (New York, NY: Springer).

[B38] Mayerson N. (2016). *Psychology Today.* New York, NY: John Thomas.

[B39] McGrath R. (2015). Character strengths in 75 nations: an update. *J. Positive Psychol.* 10 41–52. 10.1080/17439760.2014.888580

[B40] McGrath R. E. (2018). Refining our understanding of the VIA classification: reflections on papers by Han, Miller, and Snow. *J. Posit. Psychol.* 14, 41–50. 10.1080/17439760.2018.1528382

[B41] McGrath R. Greenberg M. Hall-Simmonds A. (2018). Scarecrow, Tin Woodsman, and Cowardly Lion: The Three Factor model of virtue. *J. Positive Psychol.* 13:373392.

[B42] McGrath R. E. (2019). *The VIA Assessment Suite for Adults: Development and Initial Evaluation, Revised edition.* Cincinnati, OH: VIA Institute on Character.

[B43] New Scientist (2017). *The Ethics Issue.* Netherlands: New Scientist.

[B44] Niemiec R. M. (2019a). *The Strengths-Based Workbook for Stress Relief.* Oakland, CA: New Harbinger.

[B45] Niemiec R. (2019). Six functions of character strengths for thriving at times of adversity and opportunity: a theoretical perspective. *Appl. Res. Qual. Life* 15, 551–572. 10.1007/s11482-018-9692-9692

[B46] Niemiec R. M. (2018). *Character Strengths Interventions. A Field-Guide for Practitioners.* Boston: Hogrefe.

[B47] Niemiec R. M. (2019b). Finding the golden mean: the overuse, underuse, and optimal use of character strengths. *Couns. Psychol. Q.* 10.1080/09515070.2019.1617674

[B48] Niemiec R. M. McGrath R. E. (2019). *The Power of Character Strengths: Appreciate and Ignite Your Positive Personality.* Cincinnati, OH: VIA Institute on Character.

[B49] Paldiel M. (2007). *The Righteous Among the Nations: Rescuers of Jews during the Holocaust*, 1st Edn New York, NY: Harper.

[B50] Park N. Peterson C. (2008). Positive psychology and character strengths: Application to strengths-based school counseling. *Profess. Sch. Couns.* 12 85–92. 10.5330/psc.n.2010-12.85

[B51] Park N. Peterson C. (2009). Character strengths: research and practice. *J. Coll. Character* 10:4.

[B52] Peterson C. Park N. Pole N. D’Andrea W. Seligman M. E. P. (2008). Strengths of character and posttraumatic growth. *J. Traumatic Stress* 21 214–217. 10.1002/jts.20332 18404632

[B53] Peterson C. Seligman M. (2004). *Character Strengths and Virtues: A Handbook and Classification.* New York, NY: Oxford University Press.

[B54] Peterson C. Seligman M. E. P. (2003). Character strengths before and after September 11. *Psychol. Sci.* 14 381–384. 10.1111/1467-9280.24482 12807415

[B55] Pinker S. (2011). *The Better Angels of our Nature: Why Violence has Declined.* New York, NY: Penguin Books.

[B56] Rose T. (2015). *The End of Average: How We Succeed in a World That Values Sameness.* New York, HY: Harper One.

[B57] Ruch W. Bruntsch R. Wagner L. (2017). The Role of Character Traits in Economic games. *Pers. Individ. Differ.* 108 186–190. 10.1016/j.paid.2016.12.007

[B58] Ruch W. Gander F. Platt T. Hofmann J. (2018). Team Roles: Their relationships to character strengths and job satisfaction. *J. Positive Psychol.* 13 190–199. 10.1080/17439760.2016.1257051

[B59] Ruch W. Gander F. Platt T. Hofmann J. (2016). Team roles: Their relationships to character strengths and job satisfaction. *J. Positive Psychol.* 13 190–199.

[B60] Ruch W. Gander F. Wagner L. Giuliani F. (2019). The structure of character: on the relationships between character strengths and virtues. *J. Positive Psychol.* 10.1080/17439760.2019.1689418

[B61] Ruch W. Proyer R. T. (2015). Mapping strengths into virtues: the relation of the 24 VIA-strengths to six ubiquitous virtues. *Front. Psychol.* 6:460. 10.3389/fpsyg.2015.00460 25954222 PMC4404979

[B62] Russel G. Lightman S. (2019). The human stress response. *Nat. Rev. Endocrinol.* 15 525–534. 31249398 10.1038/s41574-019-0228-0

[B63] Schnall S. Roper J. (2011). Elevation puts moral values into action. *Soc. Psychol. Pers. Sci.* 3 373–378. 10.1177/1948550611423595

[B64] Seligman M. E. P. (2002a). *Authentic Happiness: Using the New Positive Psychology to Realize Your Potential for Lasting Fulfillment.* New York, NY: Atria Paperback.

[B65] Seligman M. (2012). *Flourish: A Visionary New Understanding of Happiness and Well-Being.* New York, NY: Free Press.

[B66] Seligman M. E. P. (2002b). “Positive psychology, positive prevention, and positive therapy,” in *Handbook of Positive Psychology*, eds Snyder C. R. Lopez S. J. (Oxford University Press), 3–9.

[B67] Seligman M. E. P. Csikszentmihalyi M. (2000). Positive psychology: an introduction. *Am. Psychol.* 55 5–14. 11392865 10.1037//0003-066x.55.1.5

[B68] Shin J. Grant A. (2020). When putting work off pays off: the curvilinear relationship between procrastination and creativity. *Acad. Manag. J.* (in press). 10.5465/amj.2018.1471

[B69] Shoshani A. Slone M. (2012). Middle school transition from the strengths perspective: Young adolescents’ character strengths, subjective well-being, and school adjustment. *J. Happ. Stud.* 14 1163–1181. 10.1007/s10902-012-9374-y

[B70] Shoshani A. Slone M. (2016). The resilience function of character strengths in the face of war and protracted conflict. *Front. Psychol.* 6:2006. 10.3389/fpsyg.2015.02006 26793139 PMC4709440

[B71] Snow N. (2018). Positive psychology, the classification of character strengths and virtues, and issues of measurement. *J. Positive Psychol.* 14 20–31. 10.1080/17439760.2018.1528376

[B72] Soroka S. Fournier P. Nir L. (2019). Cross-national evidence of a negativity bias in psychophysiological reactions to news. *Proc. Natl. Acad. Sci.* 116 18888–18892. 10.1073/pnas.1908369116 31481621 PMC6754543

[B73] Vaish A. Grossmann T. Woodward A. (2008). Not all emotions are created equal: the negativity bias in social-emotional development. *Psychol. Bull.* 134:383. 10.1037/0033-2909.134.3.383 18444702 PMC3652533

[B74] Veldorale-Brogan A. Bradford K. Vail A. (2010). Marital virtues and their relationship to individual functioning, communication, and relationship adjustment. *J. Positive Psychol.* 5 281–293. 10.1080/17439760.2010.498617

[B75] Via Institute on Character (2020a). *Overview of Assessments.* Ohio: Via Institute on Character.

[B76] Via Institute on Character (2020b). *What the Research Says About Character Strengths.* Ohio: Via Institute on Character.

[B77] Wagner L. Gander F. Proyer R. T. Ruch W. (2019). Character strengths and PERMA: Investigating the relationships of character strengths with a multidimensional framework of well-being. *Appl. Res. Qual. Life* 15 307–328. 10.1007/s11482-018-9695-z

[B78] Wagner L. Ruch W. (2015). Good character at school: positive classroom behavior mediates the link between character strengths and school achievement. *Front. Psychol.* 6:610. 10.3389/fpsyg.2015.00610 26029144 PMC4432234

[B79] Waters L. (2015a). Strength-based parenting and life satisfaction in teenagers. *Adv. Soc. Sci. Res. J.* 2 158–173. 10.14738/assrj.211.1651158B173

[B80] Waters L. (2015b). The relationship between strength-based parenting with children’s stress levels and strength-based coping approaches. *Psychology* 6 689–699. 10.4236/psych.2015.66067

[B81] Waters L. E. Loton D. Jach H. K. (2019). Does strength based parenting predict academic achievement? The mediating effects of perseverance and engagement. *J. Happiness Stud.* 20 1121–1140. 10.1007/s10902-018-9983-1

[B82] Weber M. Wagner L. Ruch W. (2016). Positive feelings at school: On the relationships between students’ character strengths, school-related affect, and school functioning. *J. Happiness Stud.* 17 341–355. 10.1007/s10902-014-9597-1

[B83] Wright A. Zimmerman J. (2019). Applied ambulatory assessment: integrating idiographic and nomothetic principles of measurement. *Psychol. Assess.* 31 1467–1480. 10.1037/pas0000685 30896209 PMC6754809

[B84] Wright R. (2000). *Nonzero: The Logic of Human Destiny.* New York, NY: Random House.

